# Nonpublication Rates and Characteristics of Registered Randomized Clinical Trials in Digital Health: Cross-Sectional Analysis

**DOI:** 10.2196/11924

**Published:** 2018-12-18

**Authors:** Mustafa Al-Durra, Robert P Nolan, Emily Seto, Joseph A Cafazzo, Gunther Eysenbach

**Affiliations:** 1 Centre for Global eHealth Innovation Techna Institute University Health Network Toronto, ON Canada; 2 Institute of Health Policy, Management and Evaluation Dalla Lana School of Public Health University of Toronto Toronto, ON Canada; 3 Cardiac eHealth and Behavioral Cardiology Research Unit Peter Munk Cardiac Centre University Health Network Toronto, ON Canada; 4 Department of Psychology University of York Toronto, ON Canada; 5 Institute of Biomaterials and Biomedical Engineering University of Toronto Toronto, ON Canada

**Keywords:** clinical protocols, clinical trial, eHealth, mHealth, mobile health, publications, publication bias, randomized controlled trial, registries, telehealth, telemedicine

## Abstract

**Background:**

Clinical trials are key to advancing evidence-based medical research. The medical research literature has identified the impact of publication bias in clinical trials. Selective publication for positive outcomes or nonpublication of negative results could misdirect subsequent research and result in literature reviews leaning toward positive outcomes. Digital health trials face specific challenges, including a high attrition rate, usability issues, and insufficient formative research. These challenges may contribute to nonpublication of the trial results. To our knowledge, no study has thus far reported the nonpublication rates of digital health trials.

**Objective:**

The primary research objective was to evaluate the nonpublication rate of digital health randomized clinical trials registered in ClinicalTrials.gov. Our secondary research objective was to determine whether industry funding contributes to nonpublication of digital health trials.

**Methods:**

To identify digital health trials, a list of 47 search terms was developed through an iterative process and applied to the “Title,” “Interventions,” and “Outcome Measures” fields of registered trials with completion dates between April 1, 2010, and April 1, 2013. The search was based on the full dataset exported from the ClinlicalTrials.gov database, with 265,657 trials entries downloaded on February 10, 2018, to allow publication of studies within 5 years of trial completion. We identified publications related to the results of the trials through a comprehensive approach that included an automated and manual publication-identification process.

**Results:**

In total, 6717 articles matched the *a priori* search terms, of which 803 trials matched our latest completion date criteria. After screening, 556 trials were included in this study. We found that 150 (27%) of all included trials remained unpublished 5 years after their completion date. In bivariate analyses, we observed statistically significant differences in trial characteristics between published and unpublished trials in terms of the intervention target condition, country, trial size, trial phases, recruitment, and prospective trial registration. In multivariate analyses, differences in trial characteristics between published and unpublished trials remained statistically significant for the intervention target condition, country, trial size, trial phases, and recruitment; the odds of publication for non-US–based trials were significant, and these trials were 3.3 (95% CI 1.845-5.964) times more likely to be published than US–based trials. We observed a trend of 1.5 times higher nonpublication rates for industry-funded trials. However, the trend was not statistically significant.

**Conclusions:**

In the domain of digital health, 27% of registered clinical trials results are unpublished, which is lower than nonpublication rates in other fields. There are substantial differences in nonpublication rates between trials funded by industry and nonindustry sponsors. Further research is required to define the determinants and reasons for nonpublication and, more importantly, to articulate the impact and risk of publication bias in the field of digital health trials.

Original Paper

## Introduction

### Background

Empirical observations demonstrate that not all clinical studies successfully publish their results in peer-reviewed journals. Perhaps, the earliest indication of publication bias in the area of scientific research was in 1979 by Robert Rosenthal with the term “file drawer problem,” acknowledging the existence of selective publication bias for studies with positive and significant results [1]. A decade later, Kay Dickersin defined publication bias as “the tendency on the part of investigators, reviewers, and editors to submit or accept manuscripts for publication based on the direction or strength of the study findings.” [2]. The phenomenon of publication bias in clinical trials was attributed to the tendency of primary investigators and editors to submit or publish findings that are strong or statistically significant [3-5].

In 2008, a study of publication rates of clinical trials supporting successful new Food and Drug Administration drug applications found that over half of all the included trials were unpublished 5 years after obtaining approval from the Food and Drug Administration [6]. Similar findings were reported by other studies, indicating that half of all clinical trials remain unpublished in any peer-reviewed journal [7-9]. In 2014, two studies on discontinued randomized clinical trials reported discontinuation rates of 21% and 24.9%. This presents an ethical concern when considering the scarce research resources invested in the respective trials without the dissemination of any findings [10,11].

The registration of clinical trials, first proposed by Simes in 1986 [5], provides a means to mitigate publication bias by allowing researchers, scholars, and healthcare professionals to explore another source of trial results and information that may not be published [3-5]. It also helps identify discrepancies in primary outcome reporting by comparing primary outcome measures, as indicated in the trial protocols and published primary outcomes, which poses a key risk to the validity of trials [12-17]. During the past two decades, this proposal triggered numerous calls demanding mandatory registration of clinical trials [18-23]. In September 2004, the International Committee of Medical Journal Editors (ICMJE) mandated trial registration in a public registry at or before study enrollment as a prerequisite for publication in any of the ICMJE member journals and that the public trial registry should be publicly accessible at no charge and managed by a not-for-profit organization [24,25]. Soon thereafter, major medical journals announced the adoption of this new policy, including the British Medical Journal, the Lancet, and the Journal of Medical Internet Research [18,21,26]. In October 2008, the 7th revision of the Declaration of Helsinki was adopted by the World Medical Association’s General Assembly, with increasing emphasis on prospective registration of trials and the ethical obligation on researchers to publish their study results [27].

Since its establishment in the year 2000, the ClinicalTrials.gov website, which is maintained by the United States National Library of Medicine at the National Institutes of Health, has become the world’s largest clinical trial registry, with 286,717 registered trials, 60% of which are non-US–based as of October 11, 2018 [24,28-30].

A number of studies have analyzed and reported the characteristics of publication rates of clinical trials registered in ClinicalTrials.gov [8,9,11,31] and other data sources [6,10]. However, to our knowledge, no study has thus far analyzed and reported the characteristics of publication rates within the domain of digital health. Digital health randomized clinical trials face specific challenges, including a high attrition rate, usability issues, and insufficient prior formative research [18,32-37]. These challenges may contribute to nonpublication of trial results. This study aimed to examine the prevalence and characteristics of the nonpublication rate of digital health randomized controlled trials registered in ClinicalTrials.gov.

### Research Objectives

The primary research objective was to examine the prevalence and characteristics of the nonpublication rate among digital health randomized clinical trials registered in the ClinicalTrials.gov database. The secondary research objective was to determine whether industry funding contributes to nonpublication of trials. Considering that the ClinicalTrials.gov registry is a US–based registry including 60% of non-US–based trials, we intended to explore differences in the nonpublication rate and trial size between US- and non-US–based trials [38]. We also aimed to report outcome discrepancy between prospective and published primary outcomes of the included trials.

## Methods

### Data Source

The ClinicalTrials.gov website provides free, global open access to the online registry database through a comprehensive website search page as well as download capabilities; for example, all registration information for a given trial can be downloaded in XML format via a Web service interface. For our study, we downloaded the entire ClinicalTrials.gov online database, with 265,657 registered clinical trials entries, on February 10, 2018.

### Inclusion Criteria

The research included all eHealth-, mHealth-, telehealth-, and digital health-related randomized clinical trials that are registered in the ClinicalTrials.gov website and include any information and communication technology component, such as cellular phones, mobile phones, smart phones; devices and computer-assisted interventions; internet, online websites, and mobile applications; blogs and social media components; and emails, messages, and texts.

We also included interventional and behavioral trials with or without the results. We limited our inclusion criteria to trials with latest completion dates between April 1, 2010, and April 1, 2013. The latest date between trials’ primary completion date and completion date fields was considered the latest completion date. Details regarding the evaluation of the latest completion date of trials are described in Multimedia Appendix 1 [39,40].

### Justification of the Completion Date

Our search allowed for almost 5 years of a “publication lag period” between the stated trial completion date (up to April 1, 2013) and the search date for published reports (February 10, 2018). This strategy allowed us to account for longer publication cycles that may take up to several years, as indicated in prior studies [28]. For example, a study from the Netherlands that investigated the effects of a mobile phone app on the quality of life in patients with type 1 diabetes was published on May 11, 2015 [41], but the underlying clinical trial (NCT01444534) was first received by ClinicalTrials.gov on September 26, 2011, and the last update in ClinicalTrials.gov was made on October 23, 2012. To keep our data sample relevant, representative, and manageable, we chose to focus our study on a 3-year cross-sectional analysis for trials completed between April 2010 and April 2013.

### Exclusion Criteria

Our search excluded registered clinical trials that were not randomized or only focused on electronic record-management systems such as electronic medical records, electronic health records, and hospital information systems as well as back-end integration systems, middleware applications, and Web services. Registered clinical trials that only reported on internet, Web-based, online, and computer-based surveys as well as television or online advertisement were also excluded. In addition, the search excluded registered clinical trials that focused only on biotechnology, bioinformatics analysis, and sequencing techniques. Finally, trials on medical devices and those only related to diagnostic imaging device, computerized neuropsychological, cognition, and oxygen assessment tools were excluded.

### Search Terms

The search terms and phrases were conceptually derived from the inclusion criteria. A complete list of included search terms and phrases was developed through an iterative process (Multimedia Appendix 2 [42-52]). The following list presents the final list of the 47 search terms and phrases that were included in the search process: “smartphone,” “smart-phone,” “cellphone,” “cell-phone,” “cellular phone,” “cellular-phone,” “mobile phone,” “cell phone,” “messaging,” “sms,” “texting,” “text reminder,” “short message,” “email,” “e-mail,” “iphone,” “android,” “ipad,” “fitbit,” “on-line,” “online,” “e-Health,” “eHealth,” “mhealth,” “m-health,” “internet,” “e-therapies,” “social-media,” “social media,” “facebook,” “twitter,” “whatsapp,” “information technology,” “communication technology,” “app,” “information application,” “health application,” “mobile application,” “electronic application,” “phone application,” “touch application,” “well-being application,” “informatic,” “computer,” “digital,” “web,” and “wearable.”

### Data Extraction

#### Conditions

The “condition” field in ClinicalTrials.gov was defined as “the disease, disorder, syndrome, illness, or injury that is being studied” [53]. We analyzed and consolidated a total of 487 unique conditions of the 556 included registered randomized clinical trials into eight different groups, as reported in Table 2. Details of the condition classifications are provided in Multimedia Appendix 3 [54].

#### Discontinuation Reasons

The data exported from the ClinicalTrials.gov database includes a field “Why_Stopped” that indicates the reasons for trial discontinuation. This field is populated for trials with a withdrawn, suspended, and terminated recruitment status. We extracted and evaluated the textual content of this field as part of our recruitment analysis. Details of classification of the reasons for trial discontinuations are indicated in Multimedia Appendix 4.

#### Major Technology

We analyzed the descriptions of the 556 included randomized clinical trials to identify the major type of technology that was utilized within the respective interventions. Details of major technology classifications of the trials are indicated in Multimedia Appendix 5.

**Table 1:** Analysis of randomized clinical trials by their lead sponsor information.

Lead sponsor category (N=556)		Trials, n (%)
Foundations, Institutes, and Research Centers		72 (12.9%)
Hospitals and Medical Centers		102 (18.3%)
United States Federal Government		25 (4.5%)
University		301 (54.1%)
Other		18 (3.2%)
**Industry**		38 (6.8%)
	Insurance	6 (15.8%)
	Pharmaceuticals	2 (5.3%)
	Technology and Services	29 (76.3%)
	Telecommunication	1 (3.1%)

#### Prospective Trial Registrations

The XML data exported from the ClinicalTrials.gov database did not include an explicit field to indicate whether the trial was registered prospectively. We compared each trial’s “study_first_submitted” field to the “start_date” field in order to determine if the trial was registered prospectively or retrospectively. The “study_first_submitted” field indicates the dates when the trial’s primary investigator first submitted the trial record to ClinicalTrials.gov, whereas the “start_date” field indicates the date when the first participant was enrolled in the study [53]. We considered the registration to be prospective if the “study_first_submitted” date was before the “start_date.”

#### Reporting of Study Results

The data exported from the ClinicalTrials.gov database includes a field “Has Results” to indicate whether results have been submitted for the underlying study. The XML export of the trial metadata also includes the field “FirstReceived_Results_Date,” which is the date on which the study’s first results were received. These fields are maintained by the primary investigators of the respective trials and, in many cases, as explained in the “Limitations” section, this field is updated voluntarily by the primary investigator and seems to be inconsistent. Our analysis showed that only 61 (11%) of all included 556 randomized clinical trials reported results in the ClinicalTrials.Gov database.

#### Lead Sponsor of Trials

We defined a comprehensive and specific categorization of the funding sources of trials. We analyzed the content of the “Lead_Sponsor” field, available in trials’ XML files exported from ClinicalTrials.gov, which comprises information regarding the entity or individual that sponsors the clinical study [55]. We were able to categorize the “Lead_Sponsor” field into six different groups, with a more specific breakdown for industry sponsors (Table 1).

### Identification of Publication

We exported all the contents of the 556 included registered randomized clinical trials from the ClinicalTrials.gov website in XML format and then identified existing publications by two processes: automated and manual identification processes. The automated identification process considered all publications referenced in the trial's registry record as well as a PubMed search according to each trial’s National Clinical Trial registration number. The manual identification process was a multistep process aimed to search trial publications by key trial attributes and author details in two major bibliographic databases (PubMed and Medline) as well as the Google search engine. We only considered the results of a clinical trial to be “published” if at least one of the primary outcome measures was reported. Complete details of the publication-identification processes are described in Multimedia Appendix 6 [56-59].

## Results

### Screening Process

We exported the entire ClinlicalTrials.gov database, with 265,657 registered clinical trials entries as of February 10, 2018, into a local Structured Query Language server database. The 47 indicated search terms and phrases were then applied in the Structured Query Language server database as follows:

1. For every search term and phrase, identify matching records by the [Title] OR [Interventions] OR [Outcome Measures] fields. We identified 6717 matching trials.
2. Apply the latest completion date criteria between April 1, 2010, and April 1, 2013. We obtained 803 matching trials.
3. After screening against all inclusion and exclusion criteria, 247 registered clinical trials were excluded as per the following breakdown:
  - 149 trials were not randomized.
  - 52 trials had false-positive matching terms. For example, the registered clinical trial NCT01287377 examined the association between nicotine patch messaging and smoking cessation. The trial term “messaging” was a false-positive match to one of our search terms.
  - 17 trials were only related to computerized neuropsychological, cognition, and oxygen assessment tools.
  - 11 trials focused only on internet, Web-based, online, and computer-based surveys.
  - 9 trials were limited to the phone call intervention component.
  - 5 trials were related to scanners and diagnostic imaging devices.
  - 3 trials were related to television or online advertisement.
  - 1 trial was related to electronic medical record systems.
4. Finally, 556 studies were included after screening.

**Figure 1:**
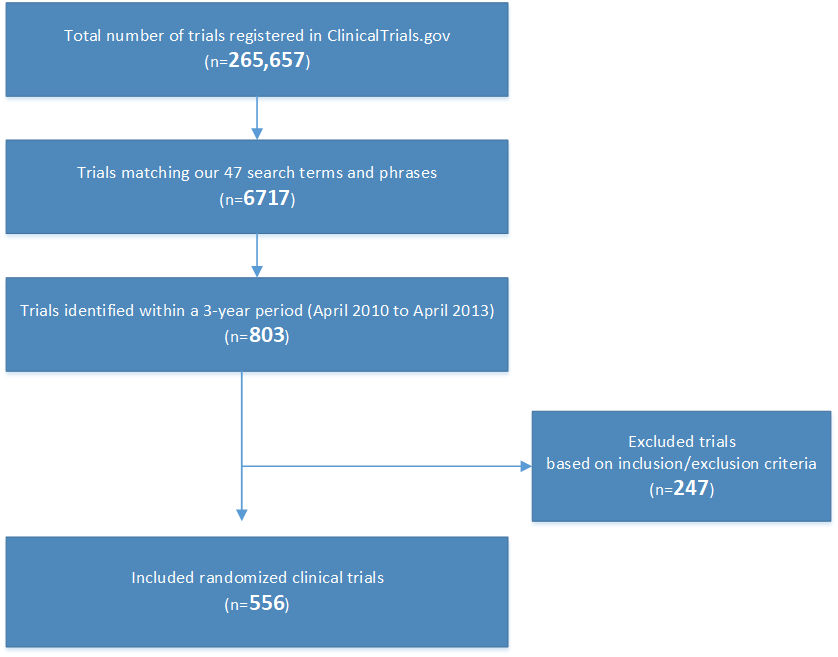
Trials included from the search results.

A summary of the search results is presented in Figure 1.

### Publication Rates

**Figure 2:**
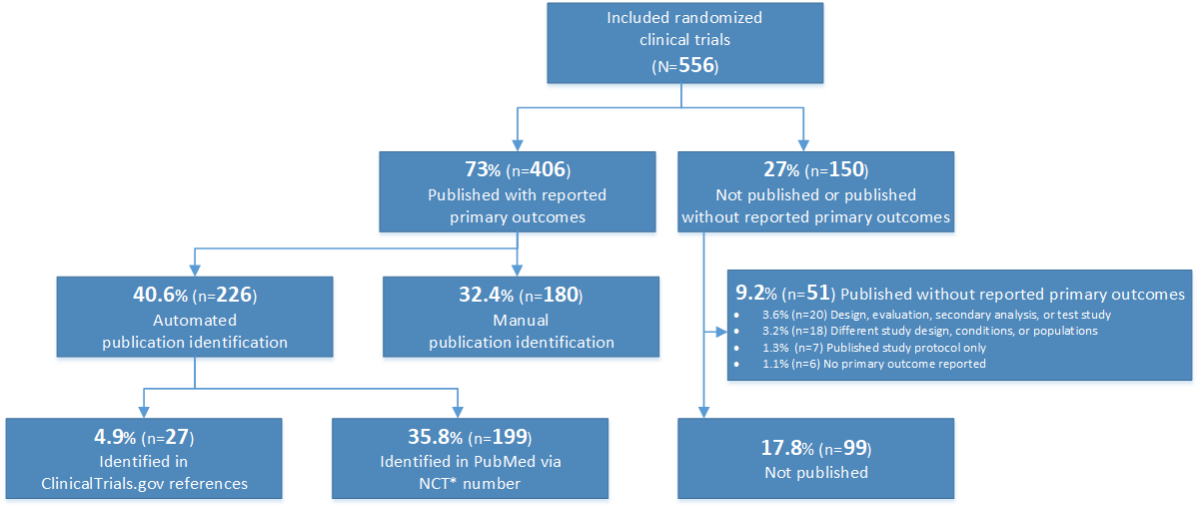
Results of the publication-identification process. *NCT: National Clinical Trial.

In summary, 406 of 556 (73%) trials were associated with identified outcome publications and 150 of 556 (27%) trials did not have any identified publications or their identified publications did not report any of their primary outcomes. Only 6 of the 556 (1.1%) published trials did not report any of the primary outcome measures indicated in the trial’s registration protocols (Figure 2).

### Analysis of Trial Characteristics

We conducted a statistical descriptive analysis, describing and summarizing the characteristics of all the 556 included registered randomized clinical trials by the following standard data elements exported from and defined by the ClinicalTrials.gov database: age group, condition, country, gender, intervention model, lead sponsor, masking, recruitment status, start date, study arms, study results, trial phase, and trial size [55]. To further our analysis, we added additional data fields that were extracted from the trial descriptions: follow-ups, latest completion date, major technology, primary outcome measure, and prospective trial registration.

We examined the relationship between trial characteristics and the nonpublication rate using bivariate and multivariate analyses. For bivariate analysis, we used the Pearson Chi-square statistical test, and for multivariate analyses, we used binary logistics regression in SPSS (IBM Corporation, Armonk, NY). The results of this analysis are depicted in Table 2.

The Pearson Chi-square test and binary logistic regression test results reported significant relationships (*P*<.05) between the nonpublication rate of trials and trial characteristics including trial condition, country, prospective registration, recruitment, trial size, and trial phases. Both tests reported no significant relationships between the nonpublication rate of trials and the age group, follow-up period, gender, intervention model, latest completion date, lead sponsor, primary outcome measures, major technology, masking, start date, study arms, and updates of trials in ClinicalTrials.gov results database.

#### Conditions

The Pearson Chi-square test results showed a significant association (*P*=.005) between the nonpublication rate and the eight different condition groups. The highest nonpublication rate was 45.2% for randomized clinical trials focusing on the “Cancer” condition. In contrast, the lowest nonpublication rate was 15.8% for randomized clinical trials focusing on “Smoking, Alcohol Consumption, Substance Abuse and Addiction” conditions. The binary logistic regression test results showed a significant association (*P*=.01) between the nonpublication rate and intervention condition groups; however, trials on cancer or addiction/smoking conditions were not a significant predictor for nonpublication (*P*=.10, odds ratio [OR]=0.414, 95% CI: 0.740-16.173 and *P*=.12, OR=3.458, 95% CI: 0.740-16.173, respectively).

#### Country

The Pearson Chi-square test results showed significant differences (*P*<.001) in the nonpublication rates between the United States and other countries; the highest nonpublication rate was observed for trials in the United States (32.8%) as compared to non-US trials. The binary logistic regression test results showed a significant association between the nonpublication rate between the US and non-US trials. The odds of publication for non-US trials were significant, and these trials were 3.3 times more likely to be published than the reference group of the US–based trials (*P*<.001, OR=3.317, 95% CI: 1.845-5.964). The global distribution of all 556 randomized clinical trials included is depicted in Multimedia Appendix 7.

#### Lead Sponsors

Only 38 (6.8%) of the 556 included registered randomized clinical trials were funded by industry sponsors. We observed a trend of 1.5 times higher nonpublication rate for industry-funded trials than non-industry-funded trials. However, this trend was not statistically significant (*P*=.07), which may be explained by the small sample size. We also found that the percentage of industry-funded trials in the US (12%) was five times higher than that in international non-US trials (2%).

#### Phases

Our Pearson Chi-square test results showed significant differences (*P*=.01) between the nonpublication rate of trials and their respective study phases. Of the 556 randomized clinical trials, 427 (76.8%) had no information reported on trial phases. For 129 (23.2%) of the randomized clinical trials that reported a study phase, phase II trials (including trials registered for both phase I and II) were most commonly reported (56 trials) and had the lowest nonpublication rate (14.3%). There were 42 phase III/IV trials (including trials registered for both phase II and III), with the highest nonpublication rate of 40.5%. The binary logistic regression test results showed a significant relationship (*P*=.004) between the nonpublication rate and trial size, and phase II trials (including trials registered for both phase I and II) were 3.9 times more likely to be published (*P*=.01, OR=3.882, 95% CI: 1.460-10.318) than other phase trials. The odds of nonpublication showed a trend towards significance for phase III/IV trials (including trials registered for both phase II and III), and these trials were 3.1 times more likely to be published (*P*=.08, OR=3.112, 95% CI: 0.876-11.054); however, the trend did not reach statistical significance.

#### Registration of Prospective Trials

We examined the relationship between prospective trial registrations and trial nonpublication rates. Results of the Pearson Chi-square test showed a statistically significant relationship (*P*=.006) between prospective trial registrations and the nonpublication rates, with higher nonpublication rates for prospectively registered trials (11.3%) than retrospectively registered trials. Our analysis also showed that only 163 (29.3%) of all our included trials were registered prospectively. We advanced our analysis to explore the impact of the 2004 ICMJE mandate and the 2008 Declaration of Helsinki on prospective trial registrations in ClinicalTrials.gov [25,27]. Results of the Pearson Chi-square test showed a statistically significant relationship (*P*<.001) between prospective trial registration and the start date of trials, with a lower number of prospective registrations reported for trials that started after 2008 (29.7%; Table 3).

**Table 2:** Relationship between the characteristics of randomized clinical trials and nonpublication rate.

Trial characteristics		Unpublished RCTs^a^/Total RCTs^a^, n (%)	P value^b^	Binary logistic regression	
				P value	Odds ratio (95% CI)
Overall		150/556 (27%)	—	—	—
**Age Group**			0.52	0.36	
	Adult	27/97 (27.8%)	—	0.47	0.689 (0.254 to 1.871)
	Adult/Senior	90/312 (28.8%)	—	0.73	0.864 (0.337 to 1.987)
	Child	0/2 (0%)	—	0.99	>999.999 (0 to >999.999)^c^
	Child/Adult	20/79 (25.3%)	—	0.29	1.738 (0.627 to 4.821)
	Child/Adult/Senior	13/66 (19.7%)	—	—	Reference
**Condition**			0.005	0.01	
	Cancer	14/31 (45.2%)	—	0.1	0.414 (0.740 to 16.173)
	Chronic pain and chronic conditions (including diabetes, asthma, and COPD^d^)	24/81 (29.6%)	—	0.52	0.752 (0.317 to 1.784)
	Heart disease, hypertension, and stroke	15/53 (28.3%)	—	0.8	1.130 (0.436 to 2.931)
	Mental health, neurodevelopmental disorders, Alzheimer, dementia, and epilepsy	14/78 (17.9%)	—	0.31	1.585 (0.648 to 3.877)
	Multiconditions	23/53 (43.4%)	—	0.11	0.480 (0.197 to 1.165)
	Obesity, weight management, nutrition, and physical activity	17/60 (28.3%)	—	0.11	2.455 (0.810 to 7.438)
	Smoking, alcohol consumption, substance abuse, and addiction	9/57 (15.8%)	—	0.12	3.458 (0.740 to 16.173)
	Others	34/143 (23.8%)	—	—	Reference
**Country**			<.001	<.001	
	Outside the United States	39/218 (17.9%)	—	<.001	3.317 (1.845 to 5.964)
	United States	111/338 (32.8%)	—	—	Reference
**Enrollment**			<.001	0.02	
	≤5th percentile (up to 26 participants)	15/29 (51.7%)	—	0.99	>999.999 (0 to >999.999)^c^
	Between the 5th and 50th percentile (between 27 and 148 participants)	58/244 (23.8%)	—	0.99	>999.999 (0 to >999.999)^c^
	Between the 50th and 95th percentile (between 149-1962 participants)	59/246 (24%)	—	0.99	>999.999 (0 to >999.999)^c^
	>95th percentile (more than 1962 participants)	8/27 (29.6%)	—	0.99	>999.999 (0 to >999.999)^c^
	Undefined	10/10 (100%)	—	—	Reference
**Follow-up period**			0.14	0.21	
	<1 month	13/56 (23.2%)	—	—	Reference
	1-3 months	34/138 (24.6%)	—	0.44	1.436 (0.574 to 3.595)
	4-6 months	32/171 (18.7%)	—	0.62	0.792 (0.314 to 1.997)
	6-12 months	45/128 (35.2%)	—	0.39	0.670 (0.272 to 1.653)
	12-24 months	12/40 (30%)	—	0.89	1.085 (0.330 to 3.570)
	>24 months	5/17 (29.4%)	—	0.9	0.908 (0.200 to 4.124)
	Undefined	9/60 (15%)	—	0.19	2.199 (0.673 to 7.185)
**Gender**			0.98	0.64	
	Both	132/491 (26.9%)	—	0.88	0.877 (0.168 to 4.567)
	Female	15/55 (27.3%)	—	0.76	1.318 (0.225 to 7.738)
	Male	3/10 (30%)	—	—	Reference
**Intervention model**			0.09	0.29	
	Single assignment	14/33 (42.4%)	—	0.99	1.475 (0.929 to 2.343)
	Crossover assignment	4/21 (19%)	—	0.99	<.001 (<.001 to >999.999)^c^
	Parallel assignment	121/464 (26.1%)	—	0.99	<.001 (<.001 to >999.999)^c^
	Factorial assignment	11/32 (34.4%)	—	0.99	<.001 (<.001 to >999.999)^c^
	Undefined	0/6 (0%)	—	—	Reference
**Latest completion date by year^d^**			0.07	0.06	
	Before 2012	63/269 (23.4%)	—	0.06	1.636 (0.987 to 2.714)
	On or after 2012	87/287 (30.3%)	—	—	Reference
**Lead sponsor – industry**			0.07	0.3	
	No	135/518 (26.1%)	—	0.3	1.609 (0.650 to 3.986)
	Yes	15/38 (39.5%)	—	—	Reference
**Major technology**			0.67	0.58	
	Computer-based intervention (offline)	27/97 (27.8%)	—	0.99	0.995 (0.119 to 8.299)
	Email notifications	7/24 (29.2%)	—	0.88	0.834 (0.082 to 8.444)
	Mobile phone application	5/14 (35.7%)	—	0.84	0.771 (0.058 to 10.204)
	Telemonitoring devices	16/64 (25%)	—	0.54	1.950 (0.226 to 16.842)
	Text messaging	9/53 (17%)	—	0.61	1.799 (0.188 to 17.215)
	Web-based intervention	84/294 (28.6%)	—	0.93	0.914 (0.114 to 7.336)
	Wii	2/10 (20%)	—	—	Reference
**Masking**			0.41	0.41	
	Open label	86/319 (26.7%)	—	0.07	12.986 (0.786 to 213.344)
	Single label	53/177 (29.9%)	—	0.12	9.041 (0.546 to 149.7930)
	Double label	7/30 (23.3%)	—	0.07	15.213 (0.781 to 296.201)
	Triple label	1/16 (6.3%)	—	0.99	>999.999 (0 to >999.999)^c^
	Quadruple label	1/7 (14.3%)	—	0.17	13.859 (0.332 to 578.089)
	Undefined	2/7 (28.6%)	—	—	Reference
**Phases**			0.01	0.004	
	0/I	5/31 (16.1%)	—	0.08	3.112 (0.876 to 11.054)
	I/II or II	8/56 (14.3%)	—	0.01	3.882 (1.460 to 10.318)
	II/III, III, or IV	17/42 (40.5%)	—	0.13	0.512 (0.217 to 1.208)
	Undefined	120/427 (28.1%)	—	—	Reference
**Primary outcome measures**			0.16	0.25	
	Adherence to treatment	11/26 (42.3%)	—	0.69	0.761 (0.202 to 2.868)
	Clinical evaluation	76/316 (24%)	—	0.42	1.386 (0.631 to 3.044)
	Drug, tobacco, and alcohol use	10/41 (24.1%)	—	0.81	0.813 (0.148 to 4.475)
	Physical activity and diet intake	9/30 (30%)	—	0.97	1.022 (0.330 to 3.161)
	Process evaluation	13/58 (22.4%)	—	0.04	2.924 (1.036 to 8.250)
	Undefined	1/3 (33.3%)	—	0.3	1.341 (0.782 to 2.297)
	Vital measurement	30/82 (36.6%)	—	—	Reference
**Prospective registration**			0.006	0.29	
	Retrospective	93/393 (23.7%)	—	0.29	1.341 (0.782 to 2.297)
	Prospective	57/163 (35%)	—	—	Reference
**Recruitment**			<.001	<.001	
	Active, not recruiting	0/1 (0%)	—	0.99	>999.999 (0 to >999.999)^c^
	Completed	105/468 (22.4%)	—	0.002	3.303 (1.564 to 6.976)
	Suspended	3/4 (75%)	—	0.21	0.188 (0.014 to 2.497)
	Terminated	11/17 (64.7%)	—	0.21	0.403 (0.098 to 1.656)
	Unknown status	21/56 (37.5%)	—	0.99	>999.999 (0 to >999.999)^c^
	Withdrawn	10/10 (100%)	—	—	Reference
**Start date by year^e^**			0.71	0.99	
	After 2008	109/413 (26.4%)	—	0.99	<.001 (<.001 to >999.999)^c^
	On or Before 2008	41/142 (28.9%)	—	0.99	<.001 (<.001 to >999.999)^c^
	Undefined	0/1 (0%)	—	—	Reference
**Study arms**			0.11	0.4	
	One	8/18 (44.4%)	—	0.17	0.240 (0.032 to 1.820)
	Two	101/410 (24.6%)	—	0.63	1.486 (0.296 to 7.459)
	Three	27/75 (36%)	—	0.74	0.756 (0.143 to 3.999)
	Four or more	11/38 (28.9%)	—	0.78	1.295 (0.219 to 7.646)
	Undefined	3/15 (20%)	—	—	Reference
**Study results reported**			0.86	0.79	
	No	133/495 (26.9%)	—	0.79	1.113 (0.512 to 2.420)
	Yes	17/61 (27.9%)	—	—	Reference

^a^RCT: randomized controlled trial.

^b^*P* value from Pearson Chi-square test.

^c^Nonconvergence was reported after 20 iterations possibly due to quasicomplete separation. Logistic regression model was not appropriate for this variable level value.

^d^The median of the latest completion date year was 2012.

^e^The cut-off point for the year of start date was set at 2008, the year when the 7th Declaration of Helsinki was adopted.

**Table 3:** Results of the Pearson Chi-square test between start date of trials and prospective trial registration.

Trial start date	Prospective trial registrations/total, n (%)	*P* value
Before or on 2008	73/142 (51.4%)	<.001
After 2008	90/414 (21.7%)	<.001

#### Recruitment

Results of the Pearson Chi-square test showed a statistically significant relationship (*P*<.001) between the trial recruitment status and nonpublication rate. Similarly, the binary logistic regression test showed a significant relationship (*P*<.001) between the trial recruitment status and nonpublication rate, and the completed trials were 3.3 times more likely to be published (*P*=.002, OR=3.303, 95% CI: 1.564-6.976). Our results also showed that discontinued trials have higher nonpublication rates than completed or active trials. We referred to trials with withdrawn, suspended, and terminated recruitment statuses as discontinued trials. We extended our analysis to explore the reasons for trial discontinuation as potential contributors to higher nonpublication rate. We examined the reasons for discontinuation of 31 trials with withdrawn, suspended, and terminated recruitment statuses among the included trials (Table 4).

**Table 4:** Summary of reasons for discontinuation.

Reason for discontinuation	Trials (N=31), n (%)
Recruitment challenges	9 (29%)
Funding challenges	6 (19%)
New study priorities	3 (10%)
Primary investigator/staff attrition	2 (6%)
Drop out	2 (6%)
Technical challenges	2 (6%)
Primary investigator/staff attrition and funding challenges	2 (6%)
Not provided	5 (16%)

Our analysis showed that recruitment and funding challenges are major factors contributing to discontinuation of trials and their nonpublication rates. Details of the classification of discontinuation reasons are provided in Multimedia Appendix 4.

#### Reporting of Study Results

Results of the Pearson Chi-square test showed no statistically significant relationship (*P*=.86) between the primary investigators who reported the results in the ClinicalTrials.gov database and the publication of trial results.

#### Time to Publication

**Figure 3:**
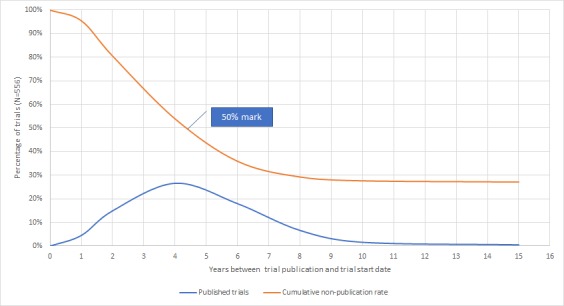
Time to publication of registered clinical trials in digital health.

We aimed to analyze the duration required to publish trial results for the 556 included trials. We measured the time to publication as the duration in years between the start date of trials and their respective publication date, which we then reported along with the number of published trials and cumulative nonpublication rates on a biyearly scale (Table 5, Figure 3).

The majority of our 556 included trials were published within 6 and 8 years of the trial’s start date (356 [64%] and 393 [70.7%], respectively). A total of 148 (26.6%) trials were published in the fourth year of the trial. We also observed that half of our included trials were published between the fourth and fifth year after the trial start date.

#### Trial Size

No enrollment values were identified for ten trials in the ClinicalTrials.gov database, and we could not identify any publications for these trials. We stratified all trials into four strata by size at the 5th, 50th, and 95th percentiles and found a statistically significant difference between the nonpublication rate of trials and trial size. The highest nonpublication rate was 51.7% for small trials that enrolled no more than 26 participants (at the 5th percentile), whereas the lowest nonpublication rate was 23.8% for trials that enrolled between 27 and 148 participants (between the 5th and 50th percentile).

The Pearson Chi-square test showed a statistically significant relationship between the nonpublication rate and trial size (*P*<.001). In addition, we found that half of the 546 randomized controlled trials that provided details of the trial size enrolled ≥148 participants (actual or intended). The cumulative enrolment in the 546 trials was 312,906 participants, split between 236,066 (75.44%) participants in published trials and 76,840 (24.56%) in unpublished trials. We found that the nonpublication rate was twice as high as that for trials below the 5th trial size percentile (≤26 participants) compared to other trials above the 5th trial size percentile (>26 participants).

**Table 5:** Analysis of trial publication cycles (duration).

Time to publication (start date to publication date), years	Published trials (N=556), n (%)	Cumulative nonpublication rate (N=556), %
2	108 (19.4%)	80.6
4	148 (26.6%)	54
6	100 (18%)	36
8	37 (6.7%)	29.3
10	9 (1.6%)	27.7
<15	3 (1%)	27.2

## Discussion

### Overview

The research literature has identified the impact and risks of publication bias for researchers, clinicians, healthcare professionals, and health policy decision makers

as well as a number of factors contributing to nonpublication and discontinuation of clinical trials [21,30,60-63]. Recruitment challenges were the most-frequently reported factor contributing to clinical trial discontinuation [10], and clinical trials with larger numbers of participants or statistically significant positive outcomes were more likely to be published [6,31,64,65]. Funding sources, study language (in particular non-English language) and study design (single-center versus multicenter studies) were also identified as contributing factors for potential bias [21,64]. Authors and primary investigators reported a lack of time as the key factor for not publishing their results in a peer-reviewed journal along with other factors such as the lack of relevance and importance of their results and disagreement with coauthors [65,66].

In the domain of digital health, we analyzed the nonpublication rate among 556 randomized clinical trials that were registered in ClinicalTrials.gov, with the latest completion date between April 2010 and April 2013. We found that 27% of all included trials remain unpublished 5 years after the latest completion date. Our finding is in line with a similar study of large randomized clinical trials, with at least 500 enrolled participants, that reported a 29% nonpublication rate [31]. However, our reported nonpublication rate (27%) was considerably less than that reported in a few other similar studies with nearly half of the trials remaining unpublished [6,7,9]. We postulate that this difference may be explained by two major factors. First, the fast-paced technology involved in digital health trials could provide more extrinsic motivation for primary investigators to share and publish their results in order to become leaders in the field and stay ahead of the digital innovation curve. Second, digital health trials are likely to be sponsored by academic entities, such as universities, hospitals, and medical and research centers, that are more disciplined and obliged by scholarly ethics to publish their results. Industry sponsors and digital technology developers, on the other hand, are likely to be more driven by the scale and opportunity in the broader digital health marketplace, beyond the realm for academia and the complexity of randomized trials design.

As part of our publication-identification process, we compared the published outcomes and primary outcomes of trials indicated in the trial registration entries in ClinicalTrials.gov. Only 6 of the 556 (1.1%) published trials did not report any of the primary outcome measures indicated in the trial registration protocols. Our finding is substantially different and should not be compared to findings from other studies that reported that 40%–62% of clinical trials had at least one change in primary outcome when comparing trial publications and protocols [12,13,15]. The difference lies in our focus on identifying trial publications with at least one reported primary outcome from the trial protocol without measuring whether all, or a subset, of the primary outcomes outlined in the trial protocol were reported or examining if secondary outcomes were reported.

We reported a statistically significant relationship between the nonpublication rate and eight different condition groups in the Pearson Chi-square test (*P*=.005) and the binary logistic regression test (*P*=.01). The highest nonpublication rate was 45.2% for randomized clinical trials focusing on the “Cancer” condition. This relative underreporting suggests challenges in conducting digital health oncology trials. These challenges align with and may be explained by findings from other studies that reported several barriers to traditional oncology trials, such as recruitment, eligibility, follow-up, and oncologist and patient attitudes [67-69]. However, we suspect that there are explicit barriers to digital health oncology trials, in particular, at the pre-enrollment and recruitment stages of the trial. Oncologists may be more inclined to enroll their patients in other traditional, nondigital health, oncology trials, where experimental drug treatment could have more tangible outcomes for their patients. Patients’ perceptions and priorities to enroll in a trial could also be influenced by the preferences of their treating oncologists. In our study, only two trials were funded by the pharmaceutical industry: This clearly small number of pharmaceutical industry-funded trials supports our postulate of explicit pre-enrollment barriers to digital health oncology trials.

We also found that half of our included trials enrolled ≥148 participants, which is similar to other findings from two different studies: 46% of trials included ≥160 participants, and 45% of trials included ≥100 participants [8,70]. On comparing trial enrollment between US–based and international randomized controlled trials, we found that US–based trials had a cumulative enrolment of 228,479 participants as compared to 48,427 participants in international trials. This finding indicates that digital health trials within the United States enroll 4.7 times more participants than international trials; this value is higher than that in all clinical trials reported in a different study, which showed that US–based trials enroll only two-thirds of the number of participants enrolled in international trials [67]. The nonpublication rate was twice as high for trials with a trial size below the 5th percentile(≤26 participants) as compared to trials with a trial size above the 5th trial size percentile (>26 participants), which is consistent with the findings of similar studies reporting that clinical trials with a larger number of participants are more likely to be published [6,31].

Randomized clinical trials are usually conducted in a series of phases, 0 to IV, to examine the intervention efficacy, safety, and adverse events over various periods and sizes of population samples [53,71-74]. However, clinical studies focusing on medical devices or behavioral interventions might not be conducted in phases and did not report information in the phase field in the ClinicalTrials.gov database [55]. The finding of our study confirms this notion, as 427 (76.8%) of the 556 included randomized clinical trials reported no information on the trial phases in the ClinicalTrials.gov database. Our results showed that phase III/IV trials have the highest nonpublication rate (40.5%) among all other phase trials and are terminated and withdrawn four times more often than other phase trials. The fact that phase III/IV trials include a large group of participants may justify the higher nonpublication, termination, and withdrawal rates when considering recruitment and attrition challenges.

In our study, we reported a statistically significant relationship between the trial recruitment status and trial nonpublication rate, and completed trials were 3.3 times more likely to be published (*P*=.002, OR=3.303, 95% CI: 1.564-6.976). Our analysis of 31 discontinued trials (trials with withdrawn, suspended, and terminated recruitment statuses) showed that enrollment and funding challenges were major contributors to the higher nonpublication rate among our included trials. This finding is in line with that of another study indicating that recruitment challenges were the most-frequently reported factor contributing to discontinuation of clinical trials [10]. Another less-frequently reported reason for discontinuation of trials is new study priorities—when the primary investigator shifts his or her priority to a new trial. The fact that a primary investigator discontinues an existing registered trial to start another new, and perhaps, similar trial questions his or her commitment to the ethics of trial registration. It is important to understand the motivation behind the discontinuation of the existing trial and the interest in starting a new trial. Primary investigators should explain if the shift in priorities to a new trial was driven by implementation challenges of the existing trial (such as insignificant outcomes and adverse events) or the research perspective of the new trial (such as a new funding or collaboration opportunity).

We analyzed the nonpublication rate with regard to the start date year of trials, stratified according to their start before or after 2008, when the 7th revision of the Declaration of Helsinki was adopted [27]. We found that the nonpublication rate for trials started in or before 2008 was 3% higher than that for trials started after 2008, although the difference was not statistically significant.

We postulate that the nonpublication rate may be higher for trials registered prospectively, as the primary investigator would register a trial before the enrollment of any participant, without knowing if the trial would be completed successfully or the results would ultimately be published. The Pearson Chi-square test showed a statistically significant relationship (*P*=.006) between prospectively registered trials and nonpublication rates, with a higher nonpublication rate for prospectively registered trials (11.3%). We also expected to see an incremental trend in the prospective registration of trials after 2008, when the 7th revision of the Declaration of Helsinki was adopted to raise awareness of prospective trial registration within the scholar community [27]. Contrary to our expectation, the Pearson Chi-square test showed a statistically significant relationship (*P*<.001) between the prospective trial registration and the trial start date, with a lower number of prospective registrations for trials starting after 2008 (29.6%). This significant decline in prospective registration, compared to the influx in retrospective registration, may be explained by the general emphasis on trial registration after 2008. It is possible that the primary investigators of unregistered trials were increasingly required to register their trials retrospectively prior to publication by the editors or the submission guidelines of the scholarly journals. However, there are two major limitations to this finding in our study: the majority (74.3%) of our included trials started after 2008, and the study scope was limited to digital health trials. These two limitations can impact the internal and external validity of our analysis to evaluate the general impact of adoption of the 7th revision of the Declaration of Helsinki on the nonpublication rate of trials and prospective trial registrations.

Most of our included trials were published within 6 to 8 years after the trial start date (356 [64%] and 393 [70.7%], respectively). We also observed that half of our included trials were published between the fourth and fifth year of the trial start date. The timelines of our findings are comparable to those of a 2007 study that analyzed time to publication of clinical trials (also measured from the start to publication date) and reported that clinical trials with statistically significant positive results were published 4-5 years after their start date, whereas trials with negative results were published in 6-8 years [75].

When we analyzed the funding sources of trials, we found that only a small number of trials (38 [6.8%] of our included trials) were funded by the industry. This finding is in contrast with the results of other studies, in which most included trials were funded by the industry. A study of delayed and nonpublication of randomized clinical trials on vaccines reported that 85% of their included trials were funded by the industry [9]. Another cross-sectional study of nonpublication of large randomized clinical trials found that 80% of the included trials were funded by the industry [31], whereas an observational study of discontinuation and nonpublication of surgical randomized controlled trials reported that 42% of the included trials were funded by the industry [11]. In our study, a majority (76.3%) of the 38 industry-sponsored trials were funded by a technology and service industry sponsor, and only two trials were funded by a pharmaceutical industry sponsor.

We observed a trend of 1.5 times higher nonpublication rates among industry-funded trials than among non-industry-funded trials. However, the trend was not statistically significant, which may be explained by the small sample size. We also found that the ratio of industry-funded trials in the United States is five times higher than that of international trials. Although these findings may be interpreted by the predominantly privately funded healthcare system in the United States, they could also be attributed to the scale of the digital health industry in the United States compared to the rest of the world, with US–based digital health startups holding 75% of the global market shares between 2013 and 2017 [76-78].

### Limitations

Despite ICMJE–mandated trial registration since 2005, not all randomized trials are registered [79]. Therefore, in practice, the proportion of unreported trials, trials that failed, and publications that did not report the primary outcomes may be different.

In this study, the ClinicalTrials.gov database was the sole data source of trial registrations. The choice was driven by feasibility challenges with limited research resources available for this study initiative and broader and global adoption of the ClinicalTrials.gov registry within the biomedical research enterprise. There are many other trials registries such as the European Clinical Trials Registry [80] and the International Standard Registered Clinical/Social Study Number (ISRCTN) registry [81]. The exclusion of all trial registries other than ClinicalTrials.gov in our analysis may have impacted the external validity (generalizability) of our findings.

Our publication-identification process was conducted between June 29, 2016, and February 10, 2018, for all included 556 randomized clinical trials. Therefore, our findings did not include studies published after February 10, 2018. This study includes trials based on their completion date and primary completion date declared in the registry record in ClinicalTrials.gov. When not provided, we considered the latest completion date as described in Multimedia Appendix 1. These criteria assume that the primary investigators and study sponsors provided and updated trial details in the ClinicalTrials.gov database. However, this is a manual and voluntarily process that may not be fully complied with, given the competing priorities and limited resources available for the primary investigators and study sponsors. These limitations may impact the generalizability of our study results.

### Conclusion

From our study of 556 randomized clinical trials in the field of digital health that are registered in the ClinicalTrials.gov database, we found that nonpublication of trials is prevalent, with almost a third (150, 27%) of all included trials remaining unpublished 5 years after their completion date. There are distinct differences in nonpublication rates between US- and non-US–based trials and according to the funding sources (industry sponsors vs non-industry sponsors). Further research is required to define the rationale behind the nonpublication rates from the perspectives of primary investigators and, more importantly, to articulate the impact and risk of publication bias in the field of digital health clinical trials. Future studies could also include nonrandomized trials such as projects published in protocols (such as JMIR Research Protocols).

It is not clear whether the research or technology failed, or if the results were disappointing and scholars did not write up a report, or if reports were rejected by journals; however, given the multitude of potential publication venues, and increased transparency in publishing, the former seems more likely. Scholarly communication is evolving, and short reports of failed trials may not always be published in peer-reviewed journals, but may be found in preprint servers. With the growing popularity of preprints, future analyses may also include searches for draft reports on preprint servers (such as preprints.jmir.org) to include unpublished reports, which may further shed light on why trials failed or remained unpublished. In the meantime, a general recommendation would be to conduct thorough formative research and pilot studies before conducting a full randomized controlled trial to reduce the risk of failure such as having insufficient power due to lack of participant engagement and nonuse attrition [82].

## Appendix

Multimedia Appendix 1 Evaluation of the latest completion date of trials.

Multimedia Appendix 2 Determination of search terms and phrases.

Multimedia Appendix 3 Classification of trial condition groups.

Multimedia Appendix 4 Classification of reasons for discontinuation of trials.

Multimedia Appendix 5 Classification of major technologies used in trials.

Multimedia Appendix 6 Identification of publications.

Multimedia Appendix 7 Global distribution of all included trials.

## Abbreviations

ICMJE: International Committee of Medical Journal Editors
OR: odds ratio
RCT: randomized controlled trial
