# Supplementary material for: Nonpublication Rates and Characteristics of Registered Randomized Clinical Trials in Digital Health: Cross-Sectional Analysis
Source: J Med Internet Res. 2018 Dec 18;20(12):e11924. doi: 10.2196/11924 (PMC6315268; doi:10.2196/11924)
Supplement: Multimedia Appendix 1 [file jmir_v20i12e11924_app1.pdf]

Appendix I - Evaluation of Trial Latest Completion Date

We evaluated the Latest Completion Date as the later of the trials' Primary Completion Date and the Completion Date fields. The Primary Completion Date is "The date on which the last participant in a clinical study was examined or received an intervention and that data for the Primary Outcome Measure were collected."[38] Whereas the Completion Date is "The date on which the final data for a clinical study were collected because the last study participant made the final visit to the study location (that is, 'last subject, last visit')."[39] Both above date fields could also be tagged as either anticipated or actual dates. "A Type menu is also included, with options Anticipated and Actual."[40] Our evaluation will consider the actual dates when provided or otherwise will consider the anticipated dates.

In cases where Completion Date is not provided, we considered the Primary Completion Date as the Latest Completion Date and vice versa. This is a best effort approach to indicate the study completion date. In the cases where both date fields, Completion Date and Primary Completion Date, are not provided, we considered the Last Updated date as the Latest Completion Date. The 'Last Updated' date field represents the most recent date when changes to a study record were submitted to ClinicalTrials.gov.[41]
