# Supplementary material for: Nonpublication Rates and Characteristics of Registered Randomized Clinical Trials in Digital Health: Cross-Sectional Analysis
Source: J Med Internet Res. 2018 Dec 18;20(12):e11924. doi: 10.2196/11924 (PMC6315268; doi:10.2196/11924)
Supplement: Multimedia Appendix 2 [file jmir_v20i12e11924_app2.pdf]

Appendix II - Determination of Search Terms and phrases

We started by summarizing all MeSH terms and authors' keywords from nine literature reviews that we identified in PubMed with the focus on the adoption and usage of eHealth, mHealth, and digital health intervention.[43-51] We then employed forward analysis by following relevant recommendations of the initial nine reviews in PubMed (first-degree recommendations) to explore other reviews' MeSH terms and authors' keywords of relevance. Lastly, we went further to a second-degree forward recommendation analysis (recommended reviews of the recommended reviews in PubMed) iteratively until we reached saturation with the terms and keywords findings. In total, we considered 16 reviews to derive our final list of MesH terms and authors' keywords.[43-53,56-60] The full list of all identified MeSH terms and authors' keywords are listed below:

Mesh Terms	[Computer-Assisted Instruction], [Evidence-Based Medicine], [Information Dissemination], [Information Services], [Internet], [Online Systems], [Therapy, Computer-Assisted], [Humans], [Counseling], [Feedback], [Health Promotion], [Life Style], [Peer Group], [Social Support], [Telecommunication], [Microcomputers], [Guideline Adherence], [Persuasive Communication], [Behavior Therapy], [Behavioral Medicine], [Age Groups], [Female], [Male], [Intervention Studies], [Motivation], [Psychology], [Social Marketing], [Chronic Disease], [Behavior], [Health Education], [Technology], [Medical Informatics], [Community Health Services], [Software], [Self Care], [Attitude to Health], [Health Status], [Attitude to Computers], [Physical Fitness], [Motor Activity], [Patient Care Planning], [Decision Support Techniques], [Computer Systems], [Access to Information]
Authors' Keywords	[e-therapy], [e-Therapies], [eTherapies], [eTherapy], [Computer-Assisted Therapy], [Computer-Assisted Therapies], [Blogging], [Blog], [Blogs], [Messaging], [Text], [Texting], [Social], [Facebook], [Twitter], [Tweets], [Persistence], [Electronic Mail], [eMail], [e-Mail], [Electronic Mails], [eMails], [e-Mails], [Promotional Health], [Life Styles], [Life Style], [Lifestyle], [Lifestyles], [Life-Styles], [Life-Style], [Behavior], [Behavioral], [Feedback], [Digital], [Interventions], [Intervention], [Promoting Health], [Persuasive], [Motivation], [Motivational], [Prevention], [Informatics], [Informatic], [Chronic], [Technology], [Biofeedback], [Wearables], [Monitoring], [Exercise]

Verification of Preliminary Set in Clinical Trials.Gov

The preliminary set of Mesh terms and authors' keywords were applied by searching the ClinlicalTrials.gov database. The Mesh terms and authors' keywords were validated on the [Title] fields of the ClinlicalTrials.gov database.

The existing search functionality in ClinlicalTrials.gov website doesn't allow limiting the search by the title field only "Search Terms—Use this field to specify words or phrases related to the studies you want to find. This performs a general search of the study information, including the title, brief description, conditions, interventions, and locations. This field is the same as the Search box at the top of every page."[61] Therefore, the complete dataset of ClinicalTrials.gov database was exported by running an unlimited search (i.e., empty string search) for all trials. The result set included a total of 265,657 trials as of February 10th, 2018. The complete results set was then imported into a local SQL Server database to enable a fully controlled and parameterized search.

After we successfully applied the Mesh terms and authors' keywords to the [Title] field search of the ClinicalTrials.gov database, we analyzed the preliminary matching trials. We considered the title field to enhance and enrich our search terms and phrases iteratively until a final list was concluded.

Throughout this process and to systematically qualify every search term and phrase, and after validating each search term and phrase against the [Title] field of the ClinicalTrials.gov database, we expanded this iterative process to validate the complete set of terms with the [Interventions] and [Outcome Measures] fields of the ClinicalTrials.gov database as well. The [Interventions] field "…specif[ies] a process or action that is the focus of a clinical study"[61], whereas the [Outcome Measures] field "…specif[ies] a measurement, decided on in advance, that is used to determine the effect of interventions on participants in a clinical trial."[61]

An example to demonstrate how the [Interventions] and [Outcome Measures] were helpful to enhance our search terms is Trial (NCT02701998) below, where the term 'mHealth' was identified in both, [Interventions] and [Outcome Measures], fields but not in the [Title] field:

NCT Trial Number	NCT02701998
Title	The Stroke and Exercise Program (StEP)
Interventions	Behavioral: mHealth-Enhance Physical Activity Intervention
Outcome Measures	Feasibility and acceptability of a mHealth-enhanced PA intervention via questionnaires…
