# Supplementary material for: Nonpublication Rates and Characteristics of Registered Randomized Clinical Trials in Digital Health: Cross-Sectional Analysis
Source: J Med Internet Res. 2018 Dec 18;20(12):e11924. doi: 10.2196/11924 (PMC6315268; doi:10.2196/11924)
Supplement: Multimedia Appendix 3 [file jmir_v20i12e11924_app3.pdf]

Appendix III – Classification of Trial Condition Groups

We analyzed and found 487 unique entries in the condition field for the 556 included registered randomized clinical trials. We identified no particular naming convention, nomenclature or coding system used for specifying the study conditions. The condition field is a free-text entry field which offers the trials' primary investigators complete flexibility to specify the trials' conditions. For example, we identified nine different terms declaring diabetes (combined for diabetes I and II) as the study's condition; "Diabetes Mellitus," "Diabetes Mellitus, Type 2," "Diabetes Mellitus Type 2," "Type 2 Diabetes," "Type 1 Diabetes," "Diabetes," "Diabetes (Insulin-requiring, Type 1 or Type 2)," "Type 2 Diabetes Mellitus," "Type II Diabetes Mellitus." Providing more systemic clinical coding and/or classification to the condition field leads to better quality information within the ClinicalTrials.gov database registry and enables more effective condition-driven secondary statistical analysis for researchers, clinicians, and healthcare professionals. The ClinicalTrials.gov database would have benefited from existing clinical condition classification systems, such as the International Classification of Diseases (ICD). However, the semantics of the condition fields in the ClinicalTrials.gov database extend to a broader range of information which may not be necessarily classified as clinical conditions: "On ClinicalTrials.gov, conditions may also include other health-related issues, such as lifespan, quality of life, and health risks"[33]. Hence, the ICD classification system may be a less appropriate option. Perhaps, a more generic classification system would better serve the context of the condition field in the ClinicalTrials.gov database. With regards to the earlier example of nine different terms for declaring diabetes (combined for diabetes I and II), we looked into the Medical Subject Headings (MeSH) browser, the U.S. National Library of Medicine (NLM) controlled vocabulary thesaurus used for indexing articles for PubMed, and we found three distinct and systemic terms, "Diabetes Mellitus," "Diabetes Mellitus, Type 1" and "Diabetes Mellitus, Type 2," that could potentially substitute the nine different terms to describe diabetes in the ClinicalTrials.gov database.[67] The Medical Subject Headings (MeSH) offers a wider range of terminology that would probably best serve the semantics of the condition field in the ClinicalTrials.gov database.
