# Supplementary material for: Nonpublication Rates and Characteristics of Registered Randomized Clinical Trials in Digital Health: Cross-Sectional Analysis
Source: J Med Internet Res. 2018 Dec 18;20(12):e11924. doi: 10.2196/11924 (PMC6315268; doi:10.2196/11924)
Supplement: Multimedia Appendix 4 [file jmir_v20i12e11924_app4.pdf]

Appendix IV – Classification of Trial Discontinuation Reasons

We have identified 7 different categories for trials' discontinuation reasons for our 31 discontinued trials, i.e., trials with withdrawn, suspended, and terminated recruitment status. The classification is described in the following table:

#	NCT Trial Number	Recruitment Status	Discontinuation Reason (Full Text from ClinicalTrials.gov)	Classification
1	NCT00641849	Terminated	Terminated by PI r/t lack of funding and retirement of study personnel.	Both Funding and PI Challenges
2	NCT01329692	Terminated	The PI has left Duke—the primary study site—and the sponsor rescinded the support.	Both Funding and PI Challenges
3	NCT01245907	Terminated	Unacceptable high drop-out rate.	Drop out
4	NCT01671033	Terminated	The drop-out rate was too high.	Drop out
5	NCT00864630	Terminated	The study upon which this project depended for subjects and the intervention was terminated prematurely due to lack of funds.	Funding Challenges
6	NCT00891631	Withdrawn	Grant application was not funded.	Funding Challenges
7	NCT00926003	Suspended	Awaiting additional funding.	Funding Challenges
8	NCT01112969	Terminated	Project ended after 3 years.	Funding Challenges
9	NCT01156324	Withdrawn	Loss of funding.	Funding Challenges
10	NCT01347528	Withdrawn	Logistic and financial accounting reasons.	Funding Challenges
11	NCT01044368	Suspended	To focus on a similar study NCT01175408 prior to recruiting participants.	New Study/Project
12	NCT01060241	Suspended	Other projects warranted more attention.	New Study/Project
13	NCT01162694	Suspended	Study was expanded and included in another study.	New Study/Project
14	NCT00973635	Withdrawn	NULL	NULL
15	NCT01067963	Terminated	NULL	NULL
16	NCT01226238	Terminated	NULL	NULL
17	NCT01226641	Terminated	NULL	NULL
18	NCT01503008	Withdrawn	NULL	NULL
19	NCT00371462	Terminated	PI no longer has an appointment and has separated from Hines VAH and project was not transferred to another PI?	PI/Staff attrition
20	NCT01439334	Withdrawn	PI left UF.	PI/Staff attrition
21	NCT00606554	Terminated	Slow recruitment of subjects.	Recruitment Challenges
22	NCT00858559	Terminated	Stopped due to low enrollment. Patients will be followed up for 3 months.	Recruitment Challenges
23	NCT00877318	Terminated	insufficient recruitment.	Recruitment Challenges
24	NCT00878202	Terminated	Insufficient recruitment.	Recruitment

				Challenges
25	NCT01007643	Terminated	Difficulty in recruitment of study participants in allotted time and funding.	Recruitment Challenges
26	NCT01228890	Withdrawn	No one enrolled.	Recruitment Challenges
27	NCT01383278	Terminated	Insufficient sample size to complete study and merged with parent grant funded study.	Recruitment Challenges
28	NCT01302938	Terminated	Stop date for randomization: 31/5/2012. Recruitment terminated due to lack of recruitment. No new safety issues were identified.	Recruitment Challenges
29	NCT01960062	Withdrawn	No potential participants met the inclusion criteria.	Recruitment Challenges
30	NCT01141868	Withdrawn	The study never opened due to technical upgrades that were needed for the SHUTi computer system.	Technical Challenges
31	NCT01532258	Withdrawn	Online program required re-design.	Technical Challenges
