# Supplementary material for: Nonpublication Rates and Characteristics of Registered Randomized Clinical Trials in Digital Health: Cross-Sectional Analysis
Source: J Med Internet Res. 2018 Dec 18;20(12):e11924. doi: 10.2196/11924 (PMC6315268; doi:10.2196/11924)
Supplement: Multimedia Appendix 5 [file jmir_v20i12e11924_app5.pdf]

Appendix V – Classification of Trial Major Technology

We have identified seven different categories for trials' major technology as follows:

1- Web-Based Intervention: We categorized 294 (53%) of the 556 included randomized clinical trials as "Web-Based Intervention" where a computer software application was utilized to connect and synchronize data with a server computer and/or in the case of a web application accessible through the internet. For example, the clinical trial (NCT01096888) applied an internet-based weight loss intervention to participants in the Women, Infants, and Children program to enhance weight loss post-partum.
2- Computer-Based Intervention (Offline): We categorized 97 (17%) of the 556 included randomized clinical trials as "Computer-Based Intervention (Offline)" where the underlying trials utilized a stand-alone software application installed offline on a computer workstation. For example, the clinical trial (NCT00947947) introduced a computerized HIV/STD prevention program delivered on a laptop computer to increase positive perceptions of condoms and increase skills to use condoms.
3- Telemedicine Device: we identified 64 (12%) of the 556 included randomized clinical trials as "Telemedicine Device" where the underlying trials used tele-medicine device in their interventions, such as the clinical trial (NCT00237692) that investigated a telemedicine intervention to improve blood pressure control in hypertensive patients.
4- Text Messaging: we identified 53(10%) of the 556 included randomized clinical trials as "Text Messaging" as these trials used texting as the major digital component in their interventions, such as the clinical trial (NCT00675389) where peer health workers send real-time text messages containing clinical and adherence data back to the central clinic to be reviewed by clinical staff.
5- Email Notifications: we identified 24 (4%) of the 556 included randomized clinical trials as "Email Notifications," as these trials used emails as the major digital component in their interventions, such as the clinical trial (NCT01876680) where participants were provided physiotherapist support through e-mails.
6- Wii: Email Notifications: we identified 10 (2%) of the 556 included randomized clinical trials as "Wii," as these trials used the Nintendo gaming console (Wii) in their interventions, such as the clinical trial (NCT01876680) where Wii was used for aerobics, strength training, and balance activities.
7- Mobile Phone Application: We also categorized 14 (3%) of the 556 included randomized clinical trials as "Mobile Phone Application" where the underlying trials utilized a mobile application delivered via smart devices, such as mobile phones and/or iPads. For example, the clinical trial (NCT01444534) investigated the utility of a diabetes mobile phone application to record the blood glucose values, dose of insulin injection, daily carbohydrate intake, amount of physical activity, and blood pressure.
