# Supplementary material for: Nonpublication Rates and Characteristics of Registered Randomized Clinical Trials in Digital Health: Cross-Sectional Analysis
Source: J Med Internet Res. 2018 Dec 18;20(12):e11924. doi: 10.2196/11924 (PMC6315268; doi:10.2196/11924)
Supplement: Multimedia Appendix 6 [file jmir_v20i12e11924_app6.pdf]

Appendix VI – Identification of Publication

We exported the complete content of the 556 included registered randomized clinical trials in XML format from the ClinicalTrials.gov website and then identified existing publications through two identification processes; automated and manual, as described in the following sections.

Automated Publication Identification Results

Every registered clinical trial in ClinicalTrials.gov has a unique registration number that starts with the three-letter prefix 'NCT' followed by eight numeric digits, for example: "NCT00906321." Publication citations and references are usually, but not always, provided in the reference element of the XML file exported from the ClinicalTrials.gov database. For example, the trial with the identifier "NCT00906321" has the following three references in the ClinicalTrials.gov-exported XML file that are not publication citations of that clinical trial, as they were external relevant references to the underlying trial's subject matter with publication years that predate the trial's start date.


```
<?xml version="1.0" encoding="UTF-8"?>
<clinical_study>
    <!--    This xml conforms to an XML Schema at:    https://clinicaltrials.gov/ct2/html/images/info/public.xsd
            and an XML DTD at:    https://clinicaltrials.gov/ct2/html/images/info/public.dtd -->
    <required_header>
        <download_date>ClinicalTrials.gov processed this data on October 14, 2016</download_date>
        <link_text>Link to the current ClinicalTrials.gov record.</link_text>
        <url>https://clinicaltrials.gov/show/NCT00906321</url>
    </required_header>
    <id_info>
        <org_study_id>999909150</org_study_id>
        <secondary_id>09-C-N150</secondary_id>
        <nct_id>NCT00906321</nct_id>
        <nct_alias>NCT00953238</nct_alias>
    </id_info>
    .
    .
    .
    <reference>
        <citation>Guttmacher AE, Collins FS. Genomic medicine--a primer. N Engl J Med. 2002 Nov 7;347(19):1512-20. Review.</citation>
        <PMID>12421895</PMID>
    </reference>
    <reference>
        <citation>Collins FS. Shattuck lecture--medical and societal consequences of the Human Genome Project. N Engl J Med. 1999 Jul 1;341(1):28-37.</citation>
        <PMID>10387940</PMID>
    </reference>
    <reference>
        <citation>Rose AL, Peters N, Shea JA, Armstrong K. Attitudes and misconceptions about predictive genetic testing for cancer risk. Community Genet. 2005;8(3):145-51.</citation>
        <PMID>16113531</PMID>
    </reference>
```


We automated the publication identification process by developing a simple computer software routine in the C#/.NET programming language to iterate over all exported 556 XML files of the included studies and generate the number of identified references and citations for every trial.

There were only 141(25%) of the 556 included registered randomized clinical trials with reported references and/or publication citations in the ClinicalTrials.gov exported XML files. When we analyzed the data, we found that only 27(5%) of the 556 included registered randomized clinical trials that cited a publication related to the registered randomized clinical trials that reported at least one of the study primary outcome measures. Our finding is six times lower than that of another study by Ross et al., where the reported references rate was 31% of all included trials.[62]

We acknowledge that the reported references and/or publication citations in the ClinicalTrials.gov database may be limited and would not suffice to identify existing publications for included trials. We postulate that the primary investigators of those trials might have indeed published the findings of their trials but didn't have the chance or interest to update their trials' records in the ClinicalTrials.gov database. In

fact, the registration and reporting requirements of the FDA Amendments Act, Section 801, also known as the FDAAA 801, has excluded feasibility trials focusing on testing prototype devices with the primary outcome measure relates to feasibility and not to health outcomes, as well as behavioral interventional trials that didn't include a drug, biologics or devices.[63,64] In September 2016, the final rule for Clinical Trials Registration and Results Information Submission (42 CFR Part 11) was released and will take effect in the ClinicalTrials.gov database as of January 18, 2017. While the final rule clarifies and expands the legal requirements for submitting results information for registered clinical trials, it does not require the primary investigators to update their trials registry with publication information.[64,65]

We, therefore, expanded our automated search approach to consider major bibliographic databases including Medline and PubMed. The clinical trials' NCT registration number is included in the Medline record when published as part of the original paper, whereas in PubMed, the NCT number is included in the Secondary Source ID (SI) field and searchable using the [si] tag.[66] For Medline, there was no web-service or any other interface to query the Medline database by means of trials' NCT numbers. Hence, we will consider Medline in the manual identification process only. For PubMed, we utilized the publicly available web-service (e-Utils) "http://eutils.ncbi.nlm.nih.gov/entrez/eutils/esearch.fcgi?db=pubmed&term=NCTxxxxxxxx [si]" to check for existing publications based on the registered clinical trials' NCT number. The API will return a structured response in an XML format to indicate whether a publication exists for the given trial. However, we noticed that some cases exist where the NCT numbers were not indicated under the Secondary Source ID (SI) field and could only be identified by expanding the search beyond the [si] field. For example, publications of the register trial number "NCT00003375" were not found when searched under the [si] field and could only be found when the search query was expanded to include all fields: "http://eutils.ncbi.nlm.nih.gov/entrez/eutils/esearch.fcgi?db=pubmed&term=NCT00003375." Therefore, we extended our software routine to loop over all 556 included registered randomized clinical trials NCT numbers, and for each NCT number, the software routine submitted two API requests to the e-Utils API: one to search only by the [si] field and the other request for a comprehensive search across all fields. When the API response is received, the software will report the count of existing publications from the two responses for every NCT Number.

We identified 228 (41%), and 233 (42%) trials of the 556 that included registered randomized clinical trials with matching publication in PubMed when searched by the Secondary Source ID (SI) [si] field and all fields, respectively. When we analyzed the finding, we found that 199 (36%) of the 556 included registered randomized clinical trials did include a reference to relevant publication in PubMed with reported study primary outcome measures. Our finding is higher than another study by Huser in 2013, that indicated that the reported publication references rate was 23% of all included trials.[26]

In total, the automated publication identification process identified publications for 226 (41%) of the 556 included registered randomized clinical trials with reported study primary outcomes measure, of which 199 were identified in PubMed and 27 ClinicalTrials.gov references.

Manual Publication Identification Process
Our automated publication identification process did not identify any publications for the remaining 330 (59%) registered randomized clinical trials. Therefore, we continued and augmented our automated publication identification process with two more systematic manual identification iterations.

First Iteration (Trials Search): included identifying existing publication by some of the trials' data fields:

1. Search Title in Medline by the trials' titles fields; [brief_title] and [official_title], If no publication identified, then:
2. Search Title in PubMed by the trials' titles fields; [brief_title] and [official_title], If no publication identified, then:
3. Google Search by the trials' titles fields; [brief_title] and [official_title]
4. If any matches are identified in any of the above three steps, verify the following data elements for the top five matches only:
    - Authors' Details: Principal Investigator and/or Responsible Party in the ClinicalTrials.gov page being one of the publication authors
    - Location Details: Institution/Facility/Affiliation (Investigators address) being the same in the publication and ClinicalTrials.gov page.
    - Study Context: Study descriptions and context as indicated in the Trial's descriptive information in Clinical Trials.gov such as the following fields as needed: Brief Summary, Detailed Description, Intervention and Enrollment.

Second Iteration (Authors Search): after completing the first iteration, and for the remaining trials with no publication identified, search by the Authors' (Primary Investigator, PI) biographies to explore any relevant publications. If no PI was provided in ClinicalTrials.gov, the search will consider the Responsible Party if provided. To identify any relevant publication for the author:

1. Google search to find the author's online profile and bibliography.
2. If not available, search by the author's name in PubMed.
3. For all listed publication and reference for the given author, search all references titles by the term 'Trial.'
4. If no publication identified, search by key terms abstracted from the trial's title, for example, in clinical trial NCT00974467, the title was "Evaluating a Website for Parents of Injured Children." The key terms were 'Parents' and 'Web.' The matching title was "After the injury: initial evaluation of a web-based intervention for parents of injured children."

The two iterations of the manual publication identification process have identified publications for 180 (32%) of the 556 included registered randomized clinical trials with a reported study primary outcomes measure.
