# Supplementary material for: Nonpublication Rates and Characteristics of Registered Randomized Clinical Trials in Digital Health: Cross-Sectional Analysis
Source: J Med Internet Res. 2018 Dec 18;20(12):e11924. doi: 10.2196/11924 (PMC6315268; doi:10.2196/11924)
Supplement: Multimedia Appendix 7 [file jmir_v20i12e11924_app7.pdf]

Appendix VII – Global Distribution of All Included Trials

Rank (By # of Trials)	Country	#RCTs (% of Total Number of RCTs)
1	United States	338 (61%)
2	Canada	34 (6%)
3	Sweden	31 (6%)
4	Germany	15 (3%)
5	United Kingdom	13 (2%)
6	Netherlands	11 (2%)
7	France	10 (2%)
8	Denmark	9 (2%)
9	Australia	7 (1%)
10	Norway	7 (1%)
11	Taiwan	5 (1%)
12	Uganda	4 (1%)
13	Spain	4 (1%)
14	Israel	4 (1%)
15	Italy	4 (1%)
16	Kenya	4 (1%)
17	Republic of Korea	4 (1%)
18	Brazil	4 (1%)
19	Finland	4 (1%)
20	China	3 (1%)
21	India	3 (1%)
22	Islamic Republic of Iran	2 (0.4%)
23	Ireland	2 (0.4%)
24	Switzerland	2 (0.4%)
25	United States & Canada	2 (0.4%)
26	United States & United Kingdom	2 (0.4%)
27	Tanzania	1 (0.2%)
28	Turkey	1 (0.2%)

29	Chile	1 (0.2%)
30	Czech Republic	1 (0.2%)
31	Cameroon	1 (0.2%)
32	Belgium	1 (0.2%)
33	Botswana	1 (0.2%)
34	Argentina	1 (0.2%)
35	Ghana	1 (0.2%)
36	Grenada	1 (0.2%)
37	Hong Kong	1 (0.2%)
38	Iceland	1 (0.2%)
39	Malaysia	1 (0.2%)
40	Pakistan	1 (0.2%)
41	Peru	1 (0.2%)
42	Philippines	1 (0.2%)
43	Poland	1 (0.2%)
44	Portugal	1 (0.2%)
45	Romania	1 (0.2%)
46	Singapore	1 (0.2%)
47	Slovenia	1 (0.2%)
48	South Africa	1 (0.2%)
49	Netherlands & Spain	1 (0.2%)
50	Mexico & Honduras	1 (0.2%)
51	United States & Puerto Rico	1 (0.2%)
52	United States & South Africa	1 (0.2%)
53	United Kingdom & Sweden	1 (0.2%)
54	Denmark & Estonia & Germany & Italy & Spain & Sweden	1 (0.2%)
